# Gene therapy-based strategies for spinal muscular atrophy—an Asia-Pacific perspective

**DOI:** 10.1186/s40348-023-00171-5

**Published:** 2023-11-15

**Authors:** Michelle A. Farrar, Loudella Calotes-Castillo, Ranil De Silva, Peter Barclay, Lani Attwood, Julie Cini, Monica Ferrie, Didu S. Kariyawasam

**Affiliations:** 1 Department of Neurology, Sydney Children’s Hospital Network, Sydney, New South Wales Australia; 2 Discipline of Paediatrics and Child Health, UNSW Medicine and Health, School of Clinical Medicine, UNSW Sydney, Sydney, New South Wales Australia; 3 Division of Paediatric Neurology, Department of Paediatrics and Neurosciences, University of the Philippines - Philippine General Hospital, Manila, Philippines; 4 Faculty of Medical Sciences, Interdisciplinary Centre for Innovation in Biotechnology and Neuroscience (ICIBN), University of Sri Jayewardenepura, Nugegoda, Sri Lanka; 5 Institute for Combinatorial Advanced Research and Education, General Sir John Kotelawala Defence University, Ratmalana, Sri Lanka; 6 Pharmacy Department, Sydney Children’s Hospitals Network, Sydney, New South Wales Australia; 7 Kids Advanced Therapeutics Programme, Sydney Children’s Hospitals Network, Kids Research, Sydney, New South Wales Australia; 8 Advocacy Beyond Borders, Melbourne, Australia

**Keywords:** Spinal muscular atrophy, Newborn screening, Gene therapy, Rare diseases, Clinical translation, Implementation, Co-design

## Abstract

Onasemnogene abeparvovec has been life-changing for children with spinal muscular atrophy (SMA), signifying the potential and progress occurring in gene- and cell-based therapies for rare genetic diseases. Hence, it is important that clinicians gain knowledge and understanding in gene therapy-based treatment strategies for SMA. In this review, we describe the development and translation of onasemnogene abeparvovec from clinical trials to healthcare practice and share knowledge on the facilitators and barriers to implementation. Rapid and accurate SMA diagnosis, awareness, and education to safely deliver gene therapy to eligible patients and access to expertise in multidisciplinary management for neuromuscular disorders are crucial for health system readiness. Early engagement and intersectoral collaboration are required to surmount complex logistical processes and develop policy, governance, and accountability. The collection and utilisation of real-world evidence are also an important part of clinical stewardship, informing ongoing improvements to care delivery and access. Additionally, a research-enabled clinical ecosystem can expand scientific knowledge and discovery to optimise future therapies and magnify health impacts. Important ethical, equity, economic, and sustainability issues are evident, for which we must connect globally.

## Background

Spinal muscular atrophy (SMA) is a monogenic neuromuscular disorder characterised by progressive degeneration of motor neurons from the brainstem and spinal cord. The pathogenesis of SMA is caused by insufficient expression of functional survival motor neuron (SMN) protein, due to biallelic pathogenic variants of the survival motor neuron 1 (*SMN1*) gene [1]. A variable number of copies of the paralogous *SMN2* gene produces minimal SMN protein, to ameliorate phenotype in a dose-dependent manner [2]. Higher *SMN2* copy numbers denote the possibility of a later onset and milder clinical presentation. The most common and severe SMA phenotype is characterised by a rapid loss of motor neurones, which may start in utero and accelerates within the first month of life, leading to substantial muscle weakness and 90% mortality by the age of 2 years [3]. Among the less severe phenotypes, infants and children living with SMA have a range of impaired motor function and accrual of neurodisability, also impacting health and quality of life.

Gene transfer therapy is one of three SMN-targeted treatment options for people with SMA resulting in improved survival and improvement or stabilisation in motor function following a one-time intravenous infusion of SMN transgenes [4]. Increased production of functional SMN protein is also accomplished by correcting *SMN2* splicing with nusinersen, a repeated intrathecally administered antisense oligonucleotide, and risdiplam, a daily oral small molecule. The lack of head-to-head trials across these three agents precludes opportunities to understand which therapeutic confers maximal benefit to a given patient, with treatment with onasemnogene abeparvovec necessitating a thorough risk-benefit analysis for each treated individual. Instead, broad predictors include age and motor function at treatment initiation, which are noted as important determinants of individual outcomes, with the youngest and those with the lowest disease burden prior to therapeutic intervention, having the most pronounced treatment responses [5]. This emphasises the unmet need for early diagnosis, facilitated by inclusion of SMA in newborn screening programmes in some countries [6, 7].

On a global scale, especially in low- and middle-income countries, there are profound disparities in obtaining a timely and accurate diagnosis, access to expert care, and limited availability of high-cost SMA therapies with orphan drug status [8, 9]. These factors have consequently segregated affected individuals into two trajectories, one where treatment ameliorates the disease burden and the other where high mortality and functional deterioration remain the experience of people and families living with SMA.

Universally, the rapid pace of clinical development, cost, and complexity of SMA gene transfer therapy has made the implementation mission challenging, with a prerequisite for early, collaborative, and continued planning, education, and capacity building, prior to effective translation into clinical practice [10]. Accordingly, with many additional gene therapy treatments in the preclinical and clinical pipelines for rare genetic diseases, it is imperative that stakeholders understand their development and share knowledge on the facilitators and barriers to implementation, thereby developing health system readiness for these advanced therapeutics to provide health benefits. As a foundation to this, an integrated and multi-sectorial approach, inclusive of patient and family advocacy groups, pharmaceutical companies, and government agencies, appears mandatory. With these challenges and opportunities in mind, we provide an Asia-Pacific perspective of the enablers and barriers for SMA gene therapy.

## The role of gene therapy in SMA

Gene therapy introduces nucleic acids (DNA or RNA) to target cells as a way of treating or altering the progression of a disease [11]. The key components of the technology include a vector (usually a replication deficient viral capsid) and enclosed expression cassette, consisting of an enhancer/promoter and transgene with associated polyadenylation signal (the latter promotes RNA translation). A range of strategies are employed, dependant on the condition and causative gene variants. For conditions caused by loss-of-function variants such as SMA, gene transfer or addition may be used to replete therapeutic protein production. Gene knockdown or silencing may be employed in conditions with a pathological gain-of-function variant.

Accordingly, onasemnogene abeparvovec is a systemic in vivo gene transfer therapy approved for SMA. Its construct includes an adeno-associated serotype 9 (AAV) viral capsid, selected for its ability to cross the blood-brain barrier and a SMN transgene (cDNA) driven by a hybrid cytomegalovirus enhancer—chicken β-actin promoter [12]. Upon transduction, the transgene is predominantly maintained as an extrachromosomal episome in the nucleus and utilises the hosts transcription and translational machinery to produce functional SMN protein.

Virus-mediated gene therapies such as onasemnogene abeparvovec are currently considered a one-time intervention as vector exposure induces an immune response to the capsid that precludes repeated treatment. Similarly, transgene expression may induce cytotoxic immune responses to the ‘foreign protein’ and be a limitation for some conditions [13]. One barrier to AAV-mediated gene therapy is pre-existing anti-capsid humoral immunity, due to exposure to wild-type AAV in the environment that can cross-react with the AAV capsid, potentially leading to its expedited removal and negating efficacy. Elevated AAV antibody concentrations in neonates and young infants may be secondary to transplacental maternal transfer, such that repeat testing to identify decreasing titres after birth may be important prior to excluding these individuals from accessing gene therapies [14].

Potential adverse and serious adverse events have been observed with gene therapies, necessitating close surveillance and knowledge of class effects and preparations to anticipate, mitigate, and manage these. More common transient effects include post infusion nausea, anorexia, vomiting, pyrexia, increases in transaminase laboratory values, decreased platelets and thrombocytopaenia, and isolated elevations in troponin without associated clinical findings [15, 16]. Rarer serious effects of thrombotic microangiopathy, hepatotoxicity, and fatal liver dysfunction have been noted [17, 18]. Systemic adjunctive steroid administration is prescribed before, during, and after (several months) gene therapy to reduce the immune response and provide anti-inflammatory and immunosuppressive effects.

## SMA gene therapy—the evolution from phase 1 to marketing approval

Gene therapy for SMA has undergone expeditious commercialisation following findings from a phase 1 open-label dose-escalation study [19]. This single-centre clinical trial involved 15 infants with SMA type 1, with weakness manifest before age 6 months and demonstrated tolerability and efficacy, and universal survival at 20 months compared with 8% in a historical control group. In addition, there were improvements in motor function, reduction in hospitalisation rates, and maintenance of bulbar function and oral feeding for the majority.

Clinical development intensified in parallel with a post marketing landscape in the USA, with further clinical trials replicating phase 1 findings and providing additional evidence of safety and efficacy [20, 21]. For presymptomatic infants, the magnitude of motor function gains was greater and nutritional, or ventilatory support was not required [22, 23]. Most presymptomatic infants achieved independent sitting, standing, and walking, some within normal developmental windows.

The conduct of these studies is informative for future gene therapy clinical trials and the expectation that clinical translation of therapeutic developments will need to be efficient and equitable across jurisdictions. Underscoring this is the requirement for the pharmaceutical and scientific community to work in tandem with clinical experts and develop processes, whereby research and drug development run in parallel to establishing multidisciplinary clinical readiness within health systems to deliver these advanced therapies safely and effectively to the community and bridge the translational gap between clinical trials and implementation within clinical practice.

As a starting point, clinical stewardship of any gene therapy programme is imperative, and as such, the timely establishment of a governance and oversight structure remains a key component of effective clinical translation of these advanced therapeutics. An early understanding of the regulatory requirements is important and varies across countries and regions. As genetically modified organisms, they have associated peculiarities that require an integrated ethical approach; therefore, pre-established health system ethics and biosafety committees may require additional education and training on these novel therapeutics and specific regulatory tools required to deliver gene therapies in a safe and effective manner and develop frameworks for assessing and mitigating uncertainties. These include but are not limited to the theoretical risks of uncontrolled expression of a delivered gene that may interfere with cell function, genomic alteration in a recipient that causes neighbouring genes to be activated or silenced, high immunogenicity, and long-term efficacy [24].

Early and wide stakeholder engagement not only facilitates gene therapy delivery but also optimises safety and care for the affected individual. By working collaboratively with pharmacy, the co-creation of standard operating procedures that align with governance structures ensures the safe transport, storage, preparation, administration, and waste management of gene therapy products (including the safe disposal of faeces and urine during the period of viral capsid shedding in the immediate post administration period). The multidisciplinary team required for optimal gene therapy delivery and management of post administration sequalae including serious adverse events is broad, reflecting the multisystemic nature of both the disease and the side effect profile of the therapeutic intervention. Key supporting departments that may require upskilling and integration into the ‘gene therapy team’ include anaesthetics, cardiology, gastroenterology, renal medicine, pathology, radiology, allied health, social work, nursing, hospital executive, and bed flow.

## Building an Australian hub and spoke model for delivering SMA gene therapy

In Australia, the clinical implementation of gene therapy for SMA was built from a neuromuscular centre of expertise and clinical trial experience at a single site, bringing together coordination of diagnosis, care and treatment, research and training collaborations, infrastructure, and resources at Sydney Children’s Hospitals Network (SCHN). The US Food and Drug Agency (FDA) approval following demonstration of safety and short-term efficacy in very small patient cohorts led to opportunities to potentially access the drug through managed access programmes and challenges for equitable and transparent premarket access within constrained capacities (Table 1). Alongside prioritising high-quality and safe patient-centred care, this first gene therapy treatment hub extended nationally, covering 7.69 million km^2^. As a hub, SCHN continues to be involved in the delivery of the AAV gene therapy and management of rare adverse events and is connected to a network of geographically dispersed centres. Together, these are involved in ensuring eligibility, informed decision-making, follow-up, and care delivery.

**Table 1:** The challenges and opportunities within the service delivery of gene therapy for paediatric centres

Challenges	Mitigators and opportunities
Uncertain potential benefits and risks to implement evidence-based care	Developing real-world evidence to evaluate the long-term efficacy and safety of gene therapy in phenotypically and genetically diverse clinical populations
Few international laboratories established for screening for AAV9 antibodies	Establishing a biobank repository to enable research in parallel with clinical implementation of gene therapy and develop ‘in country’ capacity for AAV9 antibody screening
Obtaining informed consent against a background of uncertainty	Establishing research within clinical care ecosystem so that post approval studies can address data gaps emerging from clinical trials to inform treatment decisions and guide therapeutic expectations
Navigating approvals, processes, and requirements for gene therapy administration	Early involvement and coordination of health system stakeholders with establishment of oversight committees to streamline processes alongside development of national consensus guidelines to mitigate inequities in health implementation and service provision
Developing workforce capacity, education and training for gene therapy administration	Upskilling of the workforce through knowledge exchange activities and establishment of preceptorships to disseminate practical experience of the complexities of gene therapy handling and administration
Surveillance and reporting of adverse events in the short and long term	Establishing coordinated and standardised reporting of safety and efficacy signals and collecting data as part of integrated research in clinical care model to build tools for surveillance and report adverse events to the international SMA community
Establishing infrastructure for real-world data collection and oversight	Creation of a real-world data repository, including the development of clinically meaningful biomarkers to guide therapeutic expectations and inform treatment decisions
Establishing infrastructure for the aseptic preparation of gene- and cell-based therapies	Suitable aseptic facilities for the preparation of biological products for patient administration that require specific environmental containment are not commonly available in healthcare organisations
Education and training of all stakeholders	Development of resources to improve education and provide support in decision-making for stakeholders
Anxiety and misinformation in the public domain around gene and cell therapies and the preservation or development of public trust	The inclusion of patients/families and advocates in the planning and implementation, collection of evidence and as key partners presenting to key stakeholders such as government, media and future patients and families. The opportunity is to build a group of champions from the community sector

**Fig. 1:**
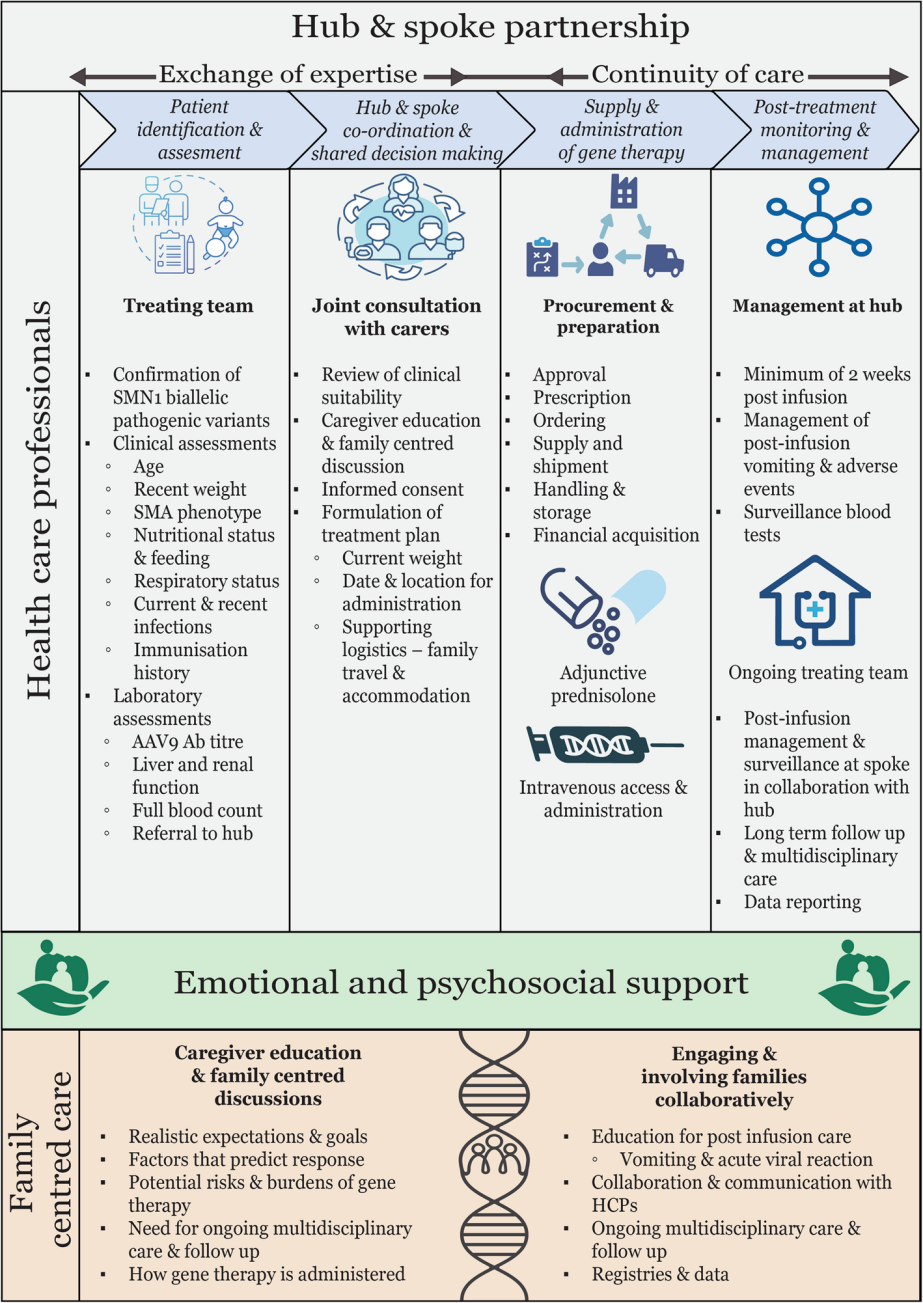
Care delivery for SMA gene therapy

Clinical protocols were established in real time, encompassing preparation, supply, administration, and monitoring (Fig. 1). A genetic diagnosis of SMA was essential, together with performing AAV9 antibody titre testing to determine whether a child with SMA was eligible for gene therapy. Strong partnerships between hub and spoke centres occurred to promote continuity of care and exchange of expertise, enabled by telehealth and joint consultations. The co-development of this model of care facilitated expansion of hub centres and mitigated health inequities due to financial and geographical barriers. The clinical suitability of gene therapy for each patient varied and was assessed in relation to all available disease-modifying treatments and ongoing provision of best practice care and support, including nutrition, respiratory, and rehabilitative care. Central to the delivery of gene therapy was family-centred care, offering emotional and psychosocial support before, during, and after gene therapy, providing clear communication and engaging and involving families collaboratively. A process for translating and utilising valuable real-world data for infants treated premarket access was developed with recognition of it being a potentially invaluable strategy to facilitate regulatory approval and enable equitable therapeutic access.

SCHN developed an Advanced Therapeutics Steering Program to help inform and address the many complexities in a considered whole organization framework. The programme is a key enabling platform for the translation of Advanced Therapeutics from preclinical to clinical research and through implementation with a focus on training and education, regulatory and sponsorship expertise, and the development and evaluation of health system models of care for implementation into clinical practice. The input from community, clinicians, individuals with expertise in early phase research ethics, clinical ethics, research sponsorship, vector technology, senior management and policy makers into risk identification, mitigation and management strategies was critical partnerships. Recognising the time critical nature of treatment and the absence of local manufacturing or storage, it was critical to enable an integrated and transparent supply chain, logistical precision and effective collaboration, to deliver a patient-centred approach.

The accredited sites to perform AAV9 antibody titre testing were in the USA and Netherlands, and processes with these international laboratories were rapidly established and then refined. The supply and administration of gene therapy entailed complex administrative agreements, accreditation, and contract management. Pharmacy played a central role in clinical stewardship, establishing processes for the procurement, handling, storage, and preparation of products. This also included facilitating chief executive sign-off for urgent high-cost therapy, which exceeded thresholds for capital purchases, and coordinating financial acquisitions and clearing. Additional pharmacy endeavours established the dosing unit ‘vg’ or vector genomes in pharmacy systems and electronic medical record dictionaries. These activities were supported by additional pharmacy resources and close coordination and communication across departments and external providers. A potential barrier was access to suitable facilities for aseptic preparation of patient doses of biological and cell-based therapies as legislated environmental containment conditions are not commonplace in most healthcare facilities. Taken together, enablers included early and continued engagement, education and awareness of both disease, technology and unmet need, a multidisciplinary approach and organisational support encompassing culture, leadership, facilities, expertise, and strong clinical governance.

## Strengthening opportunities for early diagnosis

The advent of SMA therapeutics has highlighted the importance and difficulties in achieving an early diagnosis, to enable initiation of therapy at the earliest possibility and attain greatest effect [25]. The most efficient, equitable, and cost-effective strategy is through newborn screening (NBS). Pilot studies have supported readiness and inclusion of SMA into NBS programmes, for effective translation and sustainability in clinical practice [26–28]. Ongoing endeavours focus on sharing best practices and multi-stakeholder education to facilitate expansion of SMA NBS across countries. Critical to this success are global rare disease networks, connecting centres of expertise, clinicians, policy makers and patient organisations [29]. These experiences are pertinent across rare diseases and emphasise coordinating approaches for early diagnosis in parallel with emergent gene therapies [30]. Accordingly, the opportunities and challenges related to genomic technologies are being explored internationally, with knowledge and evidence critical to inform possible expansion of NBS programmes, together with strengthening the infrastructure for NBS.

With the implementation of NBS of SMA, data gaps and ethical complexities have concomitantly emerged, including a distinct lack of knowledge on the optimal therapeutic window for individuals identified with 4 *SMN2* copies. Despite clinical recommendations from clinicians in the USA to treat infants diagnosed through NBS with SMA and 4 *SMN2* copies early, there is no international consensus [31–33]. Natural history studies of SMA and people with 4 *SMN2 copies* have shown substantial phenotypic variability in untreated individuals, ranging from onset of weakness in the first year to adulthood, albeit with functional deterioration with increasing age [34]. Several asymptomatic adults have also been described. In addition, presymptomatic clinical trials have not reported outcomes for infants with 4 *SMN2* copies to date. Taken together, these uncertainties complicate regulatory and reimbursement decisions, with inconsistent outcomes. Even in regions where treatment is accessible, not all clinicians or families choose this approach [33]. Further studies to characterise the genetic architecture of the SMN locus (e.g. long read sequencing of the *SMN2* genes) may inform strategies to accurately predict phenotype and stratify those with 4 *SMN2* copies into those requiring urgent treatment and those where long-term surveillance may be justified.

## Closing the gap: clinical research and reverse translation

**Fig. 2:**
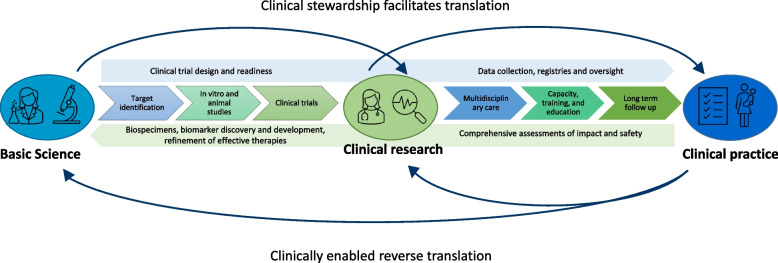
The translational research cycle: enhancing our ability to develop effective therapies and transform care

Research as part of clinical care has been fundamental to promoting the translation, efficacy, and safety of gene therapy in various SMA populations and offers opportunities to increase knowledge and develop more effective treatments (Fig. 2). Clinicians documented the natural history of SMA, validated outcome measures, and recommendations for optimal care in parallel with basic research, thereby streamlining the translation of gene therapy into clinical trials [35, 36]. The paradigm of close collaborations between basic scientists and clinicians is relevant to the development of gene therapies across rare diseases, with shared challenges of limited understanding of clinical progression, appropriate outcome measures, and small heterogeneous populations. Accordingly, selection and collection of pre-intervention real-world clinically meaningful functional, biological, and patient-reported outcome measures should occur in tandem with preclinical development, to effectively evaluate the health impact of innovative gene therapies.

Post approval real-world studies describing patient-centred and functionally meaningful outcomes for people with SMA have also facilitated formulation of individualised therapeutic expectations and clinical decision-making [37–39]. Patient age, weight, clinical status, disease severity, and duration are among the many factors that affect the safety and efficacy of gene therapy. In addition, creation of real-world evidence informs regulatory and reimbursement assessments, to improve equity of access to high-cost interventions such as gene therapy. This is especially important for this condition as market authorisations vary across health jurisdictions internationally. In Australia, the Therapeutic Goods Association approved onasemnogene abeparvovec for therapeutic use in patients younger than 9 months, whereas the US Food and Drug Agency authorised use to treat children younger than 2 years and the European Medicine Agency permits treatment up to a weight of 21 kg independent of age. The value of creating a reservoir of clinical research in tandem with gene therapy implementation is also seen from a health system perspective. From the Australian experience, a biorepository has enabled development of local AAV-9 serology testing capacity and accreditation of local laboratories, with the future potential to increase the efficiency of the drug screening pipeline. For presymptomatic SMA, infants at risk of precipitous neurodegeneration, expedient screening, and drug delivery are essential for future functional gains, such that developing capacity locally to expedite timelines remains a key concept.

Additionally, a research-enabled clinical ecosystem can power reverse translation, expanding scientific knowledge and discovery, and enhance our ability to develop effective therapies and transform care. This is exemplified by linking clinical observations with novel neurophysiological studies and biospecimen analyses to understand SMA progression, severity, and response to therapeutic intervention [40, 41]. The utility of these assessments is now being interrogated at the interface of clinical care to optimise the timing and regimen of therapeutic intervention. Repurposing of biospecimens has also enabled characterisation of the polyclonal antibody response to the AAV capsid [42]. This has the potential to inform capsid engineering and development of more efficient vectors that exhibit reduced hepatotropism and ‘off-target’ effects and an enhanced safety profile. With the total systemic dose of gene therapy calculated according to bodyweight, the ongoing development of vector technology and more specific tissue tropism may facilitate clinical delivery to heavier and older individuals and surmount manufacturing bottlenecks and costs [43].

## Challenges and opportunities for access and implementation of gene therapy for SMA across Asia Pacific

Whilst the implementation of gene therapy in high-income countries such as Australia is happening, there continues to be substantial diagnostic, therapeutic, and clinical care inequities across countries that constitute the South Asian belt. Within this region of countries where 25% of the world’s populations live, there are high-income nations with a predominantly publicly funded healthcare model (such as Australia) and developing nations that have mixed funding modalities such as the Philippines and Sri Lanka. For families of affected children with no recourse to access gene therapies, efforts to crowd source capital over months to years and across thousands of donors, and move entire families abroad, are being undertaken to access these life-changing therapeutics [44].

The challenges faced by rare disease communities is exemplified in the Philippines, where the awareness of the availability of disease-modifying treatments for SMA is still emerging. Stand for SMA Philippines, the support and advocacy group for Filipino families, recently organized and is currently pushing for urgent dialogues from all sectors (i.e. medical, government, pharmaceutical). Healthcare delivery and services provided by government do not extend to the more specialised needs of people with SMA. For example, SMA gene testing is still sent out of the country and paid out of pocket by the families, and AAV9 antibody testing is not available. International collaborations and pharmaceutical partnerships have provided free but limited gene testing for SMA for Filipino indigent patients and families. Additional barriers are encountered in establishing models of care after diagnosis and therapeutic intervention. A sole paediatric multidisciplinary neuromuscular clinic was started at the Philippine General Hospital in 2017 upon the completion of an international neuromuscular fellowship of one of the faculty of the Division of Paediatric Neurology (LCC). However, at present, this clinic has no specific funding for services and research. There are talks of including SMA in the newborn screening; however, there are no government funding or structures in place for the provision of early therapeutic intervention with gene therapies. Although the Philippines passed the Rare Disease Act of 2016 (Republic Act 10747), which is the comprehensive policy that addresses the needs of persons with rare diseases, this does not facilitate progress for SMA as in this law rare disease is defined as 1 out of 20,000 and does not include SMA.

Despite geopolitically challenging times, in countries such as Sri Lanka, efforts are underway to establish a free diagnostic service for rare disease (a substantial undertaking in areas where genetic testing is beyond many families both in availability and pricing) and to form a biobank and national registry for rare disease research [45, 46]. Establishing this clinical research structure has created a means to understand the epidemiology of SMA in the Global South, denoting a substantial number of eligible, affected individuals, as a first step towards building a case for gene therapy initiation within country. Alongside knowledge creation, human resource development remains a key area of growth for countries within Asia, with international training, global workshops, and engagement with the wider SMA expert community facilitating knowledge exchange on the implementation of gene therapy networks, within the resource framework of low- and middle-income jurisdictions [8, 47].

## Bridging the global health inequities

The significant differences in health system resources between high-, low-, and middle-income countries mean that there is no ‘one-size-fits-all’ approach for implementation of gene therapies [48]. Even so, the challenge to untie this Gordian knot may be approached by confronting common constraints and enhancing efficiencies, to achieve greater benefit for children with rare diseases.

The very high cost of SMA gene therapy (US $2.1 million for onasemnogene abeparvovec), coupled with healthcare costs for surveillance, mitigation of side effects, and supportive care, spotlights the heterogeneity of health value assessments and reimbursement structures to assess cost-effectiveness, navigate affordability, and provide patient access [49, 50]. Taken together, these preclude affected individuals in low- and middle-income countries (defined by the World Bank as nations with ≤ US $12,535 per capita) from accessing novel treatments. Budget impact tests have been introduced in some jurisdictions to help evaluate the aggregate additional cost of introduction of these therapeutics, with commercial negotiations ensuing if gene therapy exceeds a set budget threshold [51, 52]. In addition, SMA therapeutics have been considered as additions to the World Health Organization Essential Medicines list, which aim to fulfil the priority healthcare needs of populations across the 137 countries that it covers [53]. This core list of medicines is chosen specifically due to their impact on disease trajectory and are considered of high public relevance, with the intention that they are earmarked as affordable and readily available (in appropriate doses and with quality assurance) to individuals and functioning health systems. It has been proposed that inclusion of medicines into the Model List of Essential Medicines can be an important step in catalysing policy actions that may lead to more affordable pricing solutions across the world [54].

Forging rare diseases alliances between clinicians, researchers, and patient advocacy groups, to develop low-cost point-of-care technologies for diagnosis and monitoring, are enablers for timely and safe access to therapeutic interventions [8]. Increasing support for place of care manufacturing of gene therapies, whilst developing and maintaining quality standards across manufacturing sites, is also a firm objective of these global rare disease networks [55]. Concomitantly, influencing regulatory processes and creating governance and oversight education and guidance remain a key feature of successful bridging of the healthcare gap for gene therapies. Progress has been made in the international community, with a 2020 World Health Organization recommendation providing guidelines for harmonising regulatory frameworks for gene therapy products, so that regulators across low- and middle-income countries would have a standardised procedural path for assessment of gene therapy appropriateness, safety, and efficacy [56]. The World Health Organization also provides oversight and acts as an adjudicatory body for countries looking to access gene therapies [50].

Outreach and education are at the very heart of initiatives set to bridge gaps in gene therapy access, with representatives from affected local communities, technology and research sectors, government liaison and health implementation experts engaged early, to act as the powerful influencers in building readiness and infrastructure to receive and administer gene therapies [57]. Translation of resources into native languages is needed to improve community knowledge. The key role of patient and family advocacy groups cannot be underestimated or overlooked. It is the understanding of risk appetite of the patients and families, their expectations and concerns, education and information needs, support and resource requirements that inform a successful implementation [58]. A successful implementation includes patient- and family-led decision-making and an experience that ensures they feel respected and seen. By engaging early and in partnership, this ensures that full value of co-design can be implemented and experienced. It remains the patients and families together with the advocacy groups that become the strongest champions for access to and the implementation of gene and cell therapies [29, 59].

The intractable health inequities within and between communities may also be approached by taking into account the limited clinical resources, including lack of clinical experts with a comprehensive and implementational knowledge of SMA therapeutics, paucity of standardised clinical guidelines, and lack of rare disease-targeted clinical infrastructure, which precludes the best practice model of care which is founded on a multidisciplinary approach to SMA management [60, 61]. As gene therapy for SMA is a complex field, which is very demanding of material and human resources, adequate funding and strategic partnerships are imperative to provide a base to facilitate meaningful progress internationally. Establishing clinical and research partnerships from outside the developing nations remains integral to leverage expertise and design clinically translatable research pipelines. Exemplars of these challenges and novel strategies to overcome them are represented across Africa, Southeast, and Far East Asia.

The H3Africa (Human Hereditary and Health in Africa) initiative is an exemplar of systematically channelled and monitored international funding model which has brought with it capacity to establish gene therapy resources within country [62–64]. With a monetary investment of US $176 million from the Welcome Trust and National Institute of Health, the principal investigator should reside on the African continent and address problems relevant to its peoples. This has leveraged funding from non-government organisations such as the Bill and Melinda Gates Foundation. Partnerships with industry remain key drivers to facilitating access to diagnostics and therapeutics, particularly when considering efficacy of intervention in subpopulations with differing genotypes and phenotypes seen in the West. These partnerships focus on the host nation offering the industry partner well-developed expertise on these clinical subpopulations and highlight the advantage for investment in research and development. Further significant factors are the management and generation of intellectual property, which may motivate industry to partner with researchers in developing nations.

## Conclusions

The development of gene therapy for SMA represents a significant landmark not only for individuals with this disease as an exemplar but also for the therapeutic opportunities in the treatment of rare diseases. With over 1600 gene therapies in the developmental pipeline, it is anticipated that many more be implemented into clinical practice, and at an accelerated rate, recapitulating the challenges and uncertainties for translation within the rare disease domain. Building health system readiness and embedding research into clinical care as a seamless entity will underpin the efficient, safe, and equitable translation of these life-advancing and life-changing therapeutics. In tandem, the template of SMA where progressive degeneration prompts pathways for early diagnosis through NBS has laid the map for possible implementation of population screening for other conditions. This condition has therefore set the model of care where development of diagnostic and therapeutic capabilities remains equally important to fully harness the impact of advanced therapeutics such as gene therapies.

However, whilst AAV gene transfer technologies possess much anticipation and optimism for the treatment of rare diseases, challenges and uncertainties remain. Indeed, a number of disease-specific challenges have been encountered in other rare genetic diseases, including insufficient transduction, limited packaging capacity (up to 5 kb of genetic material), uncertainties over the long-term efficacy in replicating cells, and immune response. Here, novel packaging solutions, development of tissue-specific promoters to prevent overexpression of an immunogenic transgene product, and ways to dose sequentially with alternate AAV capsids, pharmacological immune modulation and even plasmapheresis have been postulated. Whilst many side effects of gene therapies can be conservatively managed through close clinical and biochemical surveillance, serious hurdles include the acute and potentially life-threatening effects, particularly observed after high-dose AAV gene therapy in older, heavier patients and in those with muscle diseases (linked to capsid, T-cell responses or the generation of a functional transgene product which has never been present or recognised by the immune system), urging careful consideration of the expected benefit and risks for patients with low physiological reserves [13, 18, 65].

Accordingly, as gene therapies become more clinically relevant, responsibilities fall onto the current clinical workforce to upskill their knowledge on the role, eligibility, delivery, support and follow-up of individuals receiving these therapeutics, to optimise efficacy and mitigate risks. Further, strengthening international collaborative links, instigating early engagement, and creating knowledge exchange opportunities among clinicians, patient organisations, policy makers and industry remain essential to drive forth equity in access, development of expertise and formation of necessary infrastructures so that the global reach of these therapeutics can be actualised.

## Data Availability

Data sharing is not applicable to this article as no datasets were generated or analysed during the current study.

## Abbreviations

AAV: Adeno-associated virus
NBS: Newborn screening
SMA: Spinal muscular atrophy
SMN: Survival motor neuron
